# *cis*-Dichloridobis(ethyl methyl sulfide-κ*S*)oxidovanadium(IV)

**DOI:** 10.1107/S1600536813006703

**Published:** 2013-03-16

**Authors:** Masatoshi Matsuura, Takashi Fujihara, Akira Nagasawa

**Affiliations:** a Department of Chemistry, Graduate School of Science and Engineering, Saitama University, Shimo-Okubo 255, Sakura-ku, Saitama 338-8570, Japan; b Comprehensive Analysis Center for Science, Saitama University, Shimo-Okubo 255, Sakura-ku, Saitama 338-8570, Japan

## Abstract

The mononuclear title complex, [VCl_2_O(C_3_H_8_S)_2_], features a V^IV^=O double bond [1.5845 (15) Å] in an overall trigonal–bipyramidal coordination environment defined by two Cl^−^ and the S atoms of two (CH_3_CH_2_)(CH_3_)S ligands. In the crystal, pairs of mol­ecules form centrosymmetric dimers *via* C—H⋯O hydrogen bonds between the methyl C—H group and the oxidovanadium O atom of a neighbouring mol­ecule.

## Related literature

For related structures, see: Azuma *et al.* (1994 ▶); Bristow *et al.* (1989 ▶); Hartung *et al.* (2005 ▶); Kakeya, Fujihara, Kasaya *et al.* (2006 ▶); Kakeya, Fujihara & Nagasawa (2006 ▶); Matsuura *et al.* (2012 ▶); Papoutsakis *et al.* (2004 ▶); Takano *et al.* (2009 ▶). For hydrogen-bonded motifs, see: Bernstein *et al.* (1995 ▶).
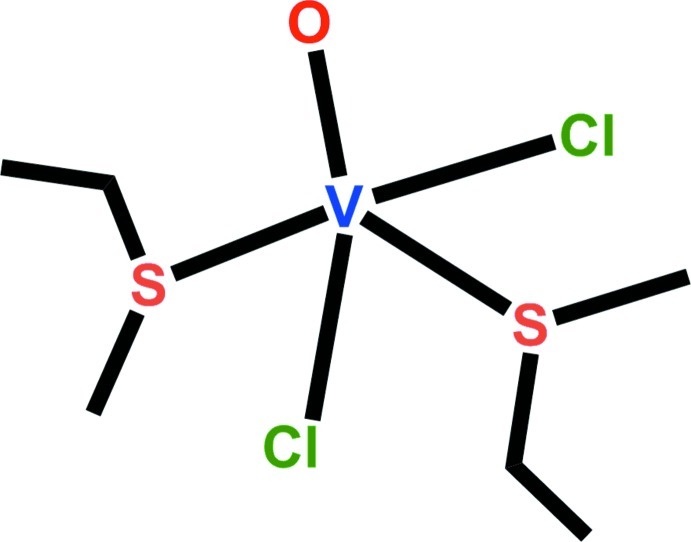


## Experimental

### 

#### Crystal data

[VCl_2_O(C_3_H_8_S)_2_]
*M* *_r_* = 290.15
Monoclinic, 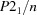

*a* = 10.503 (3) Å
*b* = 10.386 (3) Å
*c* = 11.890 (4) Å
β = 93.484 (3)°
*V* = 1294.6 (7) Å^3^
*Z* = 4
Mo *K*α radiation
μ = 1.46 mm^−1^
*T* = 150 K
0.15 × 0.13 × 0.11 mm

#### Data collection

Bruker APEXII CCD area-detector diffractometer
Absorption correction: multi-scan (*SADABS*; Bruker, 2008 ▶) *T*_min_ = 0.811, *T*_max_ = 0.856
13449 measured reflections
2646 independent reflections
2125 reflections with *I* > 2σ(*I*)
*R*_int_ = 0.059

#### Refinement

*R*[*F* ^2^ > 2σ(*F* ^2^)] = 0.031
*wR*(*F* ^2^) = 0.066
*S* = 1.24
2646 reflections
113 parameters
H-atom parameters constrained
Δρ_max_ = 0.36 e Å^−3^
Δρ_min_ = −0.37 e Å^−3^

### 

Data collection: *APEX2* (Bruker, 2008 ▶); cell refinement: *SAINT* (Bruker, 2008 ▶); data reduction: *SAINT* and *XPREP* (Bruker, 2008 ▶); program(s) used to solve structure: *SHELXS97* (Sheldrick, 2008 ▶); program(s) used to refine structure: *SHELXL97* (Sheldrick, 2008 ▶); molecular graphics: *ORTEP-3 for Windows* (Farrugia, 2012 ▶); software used to prepare material for publication: *XCIF* (Bruker, 2008 ▶).

## Supplementary Material

Crystal structure: contains datablock(s) global, I. DOI: 10.1107/S1600536813006703/cq2002sup1.cif

Structure factors: contains datablock(s) I. DOI: 10.1107/S1600536813006703/cq2002Isup2.hkl

Additional supplementary materials:  crystallographic information; 3D view; checkCIF report

## Figures and Tables

**Table 1:** Hydrogen-bond geometry (Å, °)

*D*—H⋯*A*	*D*—H	H⋯*A*	*D*⋯*A*	*D*—H⋯*A*
C1—H1⋯O1^i^	0.99	2.57	3.547 (3)	170

Symmetry code: (i) 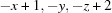.

**Fig. 1.**
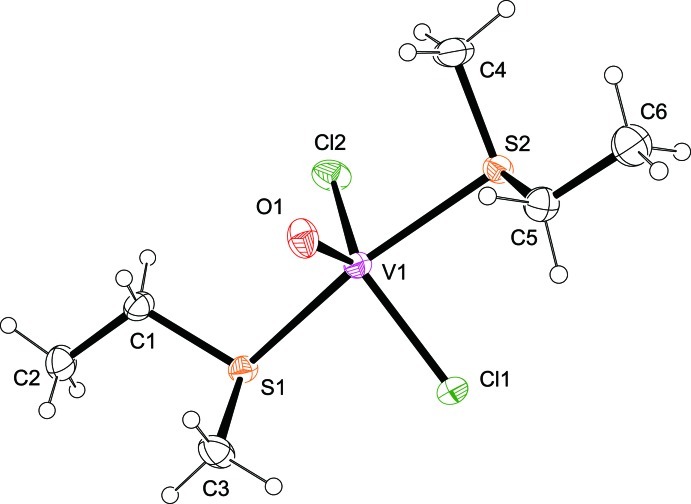
The molecular structure of the complex (I). Displacement ellipsoids are drawn at the 50% probability level.

**Fig. 2.**
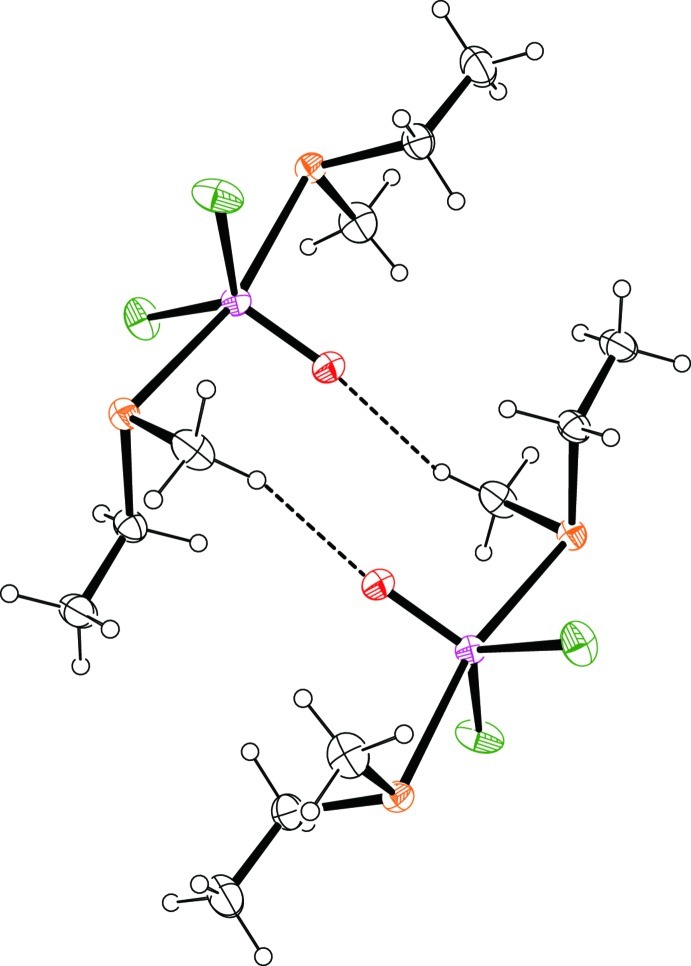
Centrosymmetric dimer of two molecules of the title compound (I) connected by mutual C—H···O hydrogen bonds (symmetry code = 1 - x, -y, 2 - z).

## supplementary crystallographic information

### Comment

The chemistry of the higher oxidation states of vanadium with neutral thioether ligands in discrete complexes remains relatively unexplored due to the instability of such complexes. Our research group has already carried out X-ray crystallographic determinations of lower oxidation state niobium complexes of the general formula [Nb_2_Cl_6_*L*^1^_2_*L*^2^] (*L*^1^= *L*^2^= tetrahydrothiophene C_4_H_8_S (THT) (Kakeya, Fujihara, Kasaya *et al.*, 2006) and dimethyl sulfide C_2_H_6_S (Kakeya, Fujihara & Nagasawa, 2006)) and (*L*^1^ = dimethyl selenide C_2_H_6_Se, *L*^2^ = C_2_H_6_S) (Matsuura *et al.*, 2012). We report here the structure of [VOCl_2_(C_3_H_8_S)_2_] (I, Scheme I). The molecule has two Cl^-^ and two EtMeS ligands (Fig. 1). Crystal structures have been reported for trigonal bipyramidal complexes formed when the water ligands in [VOCl_2_(H_2_O)_2_] are replaced by 8-hydroxyquinolinium chloride (Takano *et al.*, 2009), bis(2-(2-pyridylamino)pyridinium) dichloride (Hartung *et al.*, 2005), diethyl ether (Papoutsakis *et al.*, 2004) and benzo-15-crown-5 (Azuma *et al.*, 1994). The only trigonal bipyramidal complex containing S^2-^ reported to date is [VOCl_2_(thiourea)_2_] (II, Bristow *et al.*, 1989). In complex I, the O atom and two Cl^-^ ligands occupy the equatorial positions and the two EtMeS ligands the axial positions of a distorted trigonal bipyramid. The S2—V1—S1 angle of 166.66 (2) ^o^ deviates from the ideal value of 180 ^o^. The steric bulkness of the EtMeS ligand may be responsible for controlling the structure. The V—S distances, 2.4803 (9) and 2.4858 (9) Å, in our complex are slightly longer than that of 2.424 (1) Å in II. The average V—Cl distance and other geometrical parameters fall within the range of those in II. In the crystal, pairs of molecules form centrosymmetric *R*_2_^2^(10) dimers (Bernstein *et al.*, 1995) *via* C—H···O hydrogen bonds between the methyl C—H group and the O atom of oxidovanadium in a neighboring molecule (Fig. 2).

### Experimental

All the reactions were carried out under a dry argon atmosphere by using standard Schlenk tube techniques. Vanadium trichloride, VCl_3_ (1.0 g, 6.4 mmol), was suspended in CH_2_Cl_2_ (40 mL), and ethylmethyl sulfide (C_3_H_8_S, 1.7 mL, 19 mmol) added to the solution at room temperature. The mixture was stirred at room temperature for 2 d, during which time, a purple precipitate, probably of residual starting material, was generated gradually. This was removed by filtration. The resultant filtrate was concentrated to 5 mL before the addition of *n-*hexane (10 mL). The solution was then set aside in a freezer at 255 K. After several days, blue crystals grew in the solution. The product was too reactive with liquid water to exist at temperatures higher than 273 K even in solvent under an Ar atmosphere.

### Refinement

The H atoms were placed in calculated positions, with C—H = 0.98 (methyl) and 0.99 (methylene) Å , and refined using a riding model, with *U*_iso_(H) = 1.5.(methyl) and 1.2 *U*_eq_ (methylene).

### Crystal data

**Table:**

[VCl_2_O(C_3_H_8_S)_2_]	*F*(000) = 596
*M**_r_* = 290.15	*D*_x_ = 1.489 Mg m^−^^3^
Monoclinic, *P*2_1_/*n*	Mo *K*α radiation, λ = 0.71073 Å
Hall symbol: -P 2yn	Cell parameters from 3238 reflections
*a* = 10.503 (3) Å	θ = 2.5–27.0°
*b* = 10.386 (3) Å	µ = 1.46 mm^−^^1^
*c* = 11.890 (4) Å	*T* = 150 K
β = 93.484 (3)°	Block, blue
*V* = 1294.6 (7) Å^3^	0.15 × 0.13 × 0.11 mm
*Z* = 4	

### Data collection

**Table:**

Bruker APEXII CCD area-detector diffractometer	2646 independent reflections
Radiation source: Bruker TXS fine-focus rotating anode	2125 reflections with *I* > 2σ(*I*)
Bruker Helios multilayer confocal mirror monochromator	*R*_int_ = 0.059
Detector resolution: 8.333 pixels mm^-1^	θ_max_ = 26.4°, θ_min_ = 2.5°
φ and ω scans	*h* = −13→13
Absorption correction: multi-scan (*SADABS*; Bruker, 2008)	*k* = −12→12
*T*_min_ = 0.811, *T*_max_ = 0.856	*l* = −14→14
13449 measured reflections	

### Refinement

**Table:**

Refinement on *F*^2^	Primary atom site location: structure-invariant direct methods
Least-squares matrix: full	Secondary atom site location: difference Fourier map
*R*[*F*^2^ > 2σ(*F*^2^)] = 0.031	Hydrogen site location: inferred from neighbouring sites
*wR*(*F*^2^) = 0.066	H-atom parameters constrained
*S* = 1.24	*w* = 1/[σ^2^(*F*_o_^2^) + (0.*P*)^2^] where *P* = (*F*_o_^2^ + 2*F*_c_^2^)/3
2646 reflections	(Δ/σ)_max_ < 0.001
113 parameters	Δρ_max_ = 0.36 e Å^−^^3^
0 restraints	Δρ_min_ = −0.37 e Å^−^^3^

### Special details

**Table:**

Geometry. All esds (except the esd in the dihedral angle between two l.s. planes) are estimated using the full covariance matrix. The cell esds are taken into account individually in the estimation of esds in distances, angles and torsion angles; correlations between esds in cell parameters are only used when they are defined by crystal symmetry. An approximate (isotropic) treatment of cell esds is used for estimating esds involving l.s. planes.
Refinement. Refinement of F^2^ against ALL reflections. The weighted R-factor wR and goodness of fit S are based on F^2^, conventional R-factors R are based on F, with F set to zero for negative F^2^. The threshold expression of F^2^ > 2sigma(F^2^) is used only for calculating R-factors(gt) etc. and is not relevant to the choice of reflections for refinement. R-factors based on F^2^ are statistically about twice as large as those based on F, and R- factors based on ALL data will be even larger.

### Fractional atomic coordinates and isotropic or equivalent isotropic displacement parameters (Å^2^)

**Table:**

	*x*	*y*	*z*	*U*_iso_*/*U*_eq_	
V1	0.29967 (3)	0.19666 (4)	0.98439 (3)	0.02823 (12)	
Cl1	0.08892 (5)	0.14880 (7)	0.95318 (5)	0.0513 (2)	
Cl2	0.38968 (6)	0.37705 (6)	1.06207 (5)	0.04726 (18)	
S1	0.27796 (5)	0.11532 (6)	1.17891 (4)	0.03144 (15)	
S2	0.28225 (5)	0.31343 (6)	0.80213 (5)	0.03179 (15)	
C1	0.4391 (2)	0.1050 (2)	1.24240 (17)	0.0361 (6)	
H1A	0.4932	0.0586	1.1905	0.043*	
H1B	0.4740	0.1931	1.2527	0.043*	
C2	0.4459 (2)	0.0369 (3)	1.35491 (17)	0.0464 (7)	
H2A	0.3901	0.0805	1.4059	0.070*	
H2B	0.5339	0.0386	1.3874	0.070*	
H2C	0.4182	−0.0526	1.3444	0.070*	
C3	0.2416 (2)	−0.0523 (2)	1.15743 (19)	0.0467 (6)	
H3A	0.3105	−0.0935	1.1186	0.070*	
H3B	0.1613	−0.0607	1.1115	0.070*	
H3C	0.2332	−0.0941	1.2305	0.070*	
C4	0.4450 (2)	0.3479 (3)	0.7720 (2)	0.0492 (7)	
H4A	0.4942	0.2677	0.7732	0.074*	
H4B	0.4826	0.4070	0.8291	0.074*	
H4C	0.4468	0.3878	0.6974	0.074*	
C5	0.2422 (2)	0.1881 (2)	0.70034 (18)	0.0392 (6)	
H5A	0.3071	0.1190	0.7085	0.047*	
H5B	0.1588	0.1503	0.7169	0.047*	
C6	0.2351 (3)	0.2359 (3)	0.5799 (2)	0.0544 (7)	
H6A	0.1737	0.3068	0.5718	0.082*	
H6B	0.2075	0.1655	0.5292	0.082*	
H6C	0.3194	0.2662	0.5606	0.082*	
O1	0.39676 (14)	0.09120 (15)	0.94548 (12)	0.0415 (4)	

### Atomic displacement parameters (Å^2^)

**Table:**

	*U*^11^	*U*^22^	*U*^33^	*U*^12^	*U*^13^	*U*^23^
V1	0.02533 (19)	0.0281 (2)	0.0312 (2)	−0.00192 (16)	0.00131 (15)	0.00101 (16)
Cl1	0.0309 (3)	0.0701 (5)	0.0518 (4)	−0.0170 (3)	−0.0072 (3)	0.0221 (3)
Cl2	0.0595 (4)	0.0392 (4)	0.0433 (3)	−0.0187 (3)	0.0047 (3)	−0.0057 (3)
S1	0.0290 (3)	0.0353 (4)	0.0302 (3)	−0.0030 (3)	0.0034 (2)	−0.0008 (2)
S2	0.0319 (3)	0.0292 (4)	0.0345 (3)	−0.0001 (3)	0.0044 (2)	0.0039 (2)
C1	0.0298 (11)	0.0464 (16)	0.0317 (12)	−0.0050 (11)	−0.0007 (9)	−0.0011 (11)
C2	0.0441 (14)	0.0599 (19)	0.0344 (13)	0.0017 (13)	−0.0029 (11)	0.0058 (12)
C3	0.0614 (16)	0.0385 (16)	0.0391 (14)	−0.0180 (13)	−0.0068 (12)	0.0046 (12)
C4	0.0378 (14)	0.0590 (19)	0.0517 (15)	−0.0153 (13)	0.0097 (12)	0.0001 (13)
C5	0.0410 (13)	0.0388 (16)	0.0376 (13)	−0.0033 (11)	0.0019 (11)	−0.0036 (11)
C6	0.0561 (16)	0.070 (2)	0.0371 (14)	−0.0027 (15)	0.0041 (12)	−0.0044 (13)
O1	0.0471 (9)	0.0402 (11)	0.0374 (9)	0.0140 (8)	0.0031 (7)	0.0023 (7)

### Geometric parameters (Å, º)

**Table:**

V1—O1	1.5845 (15)	C2—H2C	0.9800
V1—Cl2	2.2695 (9)	C3—H3A	0.9800
V1—Cl1	2.2769 (9)	C3—H3B	0.9800
V1—S2	2.4803 (9)	C3—H3C	0.9800
V1—S1	2.4858 (9)	C4—H4A	0.9800
S1—C3	1.797 (3)	C4—H4B	0.9800
S1—C1	1.814 (2)	C4—H4C	0.9800
S2—C4	1.804 (2)	C5—C6	1.513 (3)
S2—C5	1.810 (2)	C5—H5A	0.9900
C1—C2	1.511 (3)	C5—H5B	0.9900
C1—H1A	0.9900	C6—H6A	0.9800
C1—H1B	0.9900	C6—H6B	0.9800
C2—H2A	0.9800	C6—H6C	0.9800
C2—H2B	0.9800		
			
O1—V1—Cl2	115.47 (7)	H2A—C2—H2C	109.5
O1—V1—Cl1	115.97 (7)	H2B—C2—H2C	109.5
Cl2—V1—Cl1	128.56 (3)	S1—C3—H3A	109.5
O1—V1—S2	95.60 (6)	S1—C3—H3B	109.5
Cl2—V1—S2	87.64 (3)	H3A—C3—H3B	109.5
Cl1—V1—S2	86.85 (2)	S1—C3—H3C	109.5
O1—V1—S1	97.69 (6)	H3A—C3—H3C	109.5
Cl2—V1—S1	87.79 (3)	H3B—C3—H3C	109.5
Cl1—V1—S1	86.23 (2)	S2—C4—H4A	109.5
S2—V1—S1	166.66 (2)	S2—C4—H4B	109.5
C3—S1—C1	100.77 (12)	H4A—C4—H4B	109.5
C3—S1—V1	103.16 (8)	S2—C4—H4C	109.5
C1—S1—V1	105.74 (7)	H4A—C4—H4C	109.5
C4—S2—C5	101.24 (11)	H4B—C4—H4C	109.5
C4—S2—V1	104.47 (9)	C6—C5—S2	113.16 (18)
C5—S2—V1	103.56 (8)	C6—C5—H5A	108.9
C2—C1—S1	112.89 (15)	S2—C5—H5A	108.9
C2—C1—H1A	109.0	C6—C5—H5B	108.9
S1—C1—H1A	109.0	S2—C5—H5B	108.9
C2—C1—H1B	109.0	H5A—C5—H5B	107.8
S1—C1—H1B	109.0	C5—C6—H6A	109.5
H1A—C1—H1B	107.8	C5—C6—H6B	109.5
C1—C2—H2A	109.5	H6A—C6—H6B	109.5
C1—C2—H2B	109.5	C5—C6—H6C	109.5
H2A—C2—H2B	109.5	H6A—C6—H6C	109.5
C1—C2—H2C	109.5	H6B—C6—H6C	109.5
			
O1—V1—S1—C3	−51.05 (11)	Cl1—V1—S2—C4	−168.84 (9)
Cl2—V1—S1—C3	−166.45 (9)	S1—V1—S2—C4	132.35 (13)
Cl1—V1—S1—C3	64.67 (9)	O1—V1—S2—C5	52.59 (10)
S2—V1—S1—C3	123.54 (13)	Cl2—V1—S2—C5	167.95 (8)
O1—V1—S1—C1	54.33 (10)	Cl1—V1—S2—C5	−63.22 (8)
Cl2—V1—S1—C1	−61.07 (9)	S1—V1—S2—C5	−122.02 (13)
Cl1—V1—S1—C1	170.05 (9)	C3—S1—C1—C2	−63.62 (19)
S2—V1—S1—C1	−131.08 (13)	V1—S1—C1—C2	−170.74 (16)
O1—V1—S2—C4	−53.04 (11)	C4—S2—C5—C6	−68.07 (19)
Cl2—V1—S2—C4	62.33 (9)	V1—S2—C5—C6	−176.14 (16)

### Hydrogen-bond geometry (Å, º)

**Table:**

*D*—H···*A*	*D*—H	H···*A*	*D*···*A*	*D*—H···*A*
C1—H1···O1^i^	0.99	2.57	3.547 (3)	170

Symmetry code: (i) −*x*+1, −*y*, −*z*+2.
